# Progress in CSF biomarker discovery in sCJD

**DOI:** 10.18632/oncotarget.13998

**Published:** 2016-12-16

**Authors:** Franc Llorens, Matthias Schmitz, Inga Zerr

**Affiliations:** ^1^ Department of Neurology, University Medicine Goettingen, Germany and German Center for Neurodegenerative Diseases, Goettingen, Germany

**Keywords:** cerebrospinal fluid, sporadic Creutzfeldt-Jakob disease, biomarkers, prion protein, differential diagnosis, Neuroscience

Sporadic Creutzfeldt-Jakob disease (sCJD) is the most prevalent form of human prion diseases caused by the misfolding of the cellular prion protein (PrPc) into its pathogenic form (PrPSc). It is clinically characterized by rapidly progressive dementia leading to the death of the patient usually in less than one year after disease onset. In the brain tissue, spongiform vacuolation, massive neuronal loss, gliosis and the presence of prion protein depositions are classical neuropathological hallmarks. The diagnosis of sCJD is usually achieved by a combination of clinical evaluation, magnetic resonance imaging, electroencephalogram and cerebrospinal fluid (CSF) tests. However, a definite diagnostic can only be attained by the detection of PrPSc in a brain biopsy or autopsy.

Since the discovery of elevated tau and the presence of 14-3-3 proteins in the CSF of sCJD patients, great efforts have been undertaken to refine quantification methods and discover new biomarker approaches of clinical relevance in the diagnosis of sCJD. The importance of CSF tests for surrogate biomarkers in the differential diagnostic context relies on the partial clinical overlap among different types of dementias regarding cognitive decline, behavioural, psychiatric and motor symptoms, which makes clinical diagnosis challenging. In this regard, treatable conditions misdiagnosed as sCJD have been broadly reported [1]. A major drawback in the use of CSF-based approaches is the lack of studies including sCJD samples in the differential diagnostic context, where multiple neurological and neurodegenerative conditions are assessed. As a consequence, it becomes difficult to estimate the true accuracy of sCJD biomarkers for clinical practise.

Recently, we analysed a broad range of CSF samples from different aetiologies reporting a very high diagnostic accuracy of the p-tau/tau ratio in the correct identification of sCJD cases, reaching a sensitivity of 99% and a specificity of 97% when compared to controls [2]. The reported area under the curve values are higher than 0.9 when comparing sCJD versus controls and other neurodegenerative dementias, which is close to an absolute discrimination. Although the potential clinical application of p-tau/tau ratio as sCJD biomarker was previously described, three main conclusions can be drawn from this study. First, p-tau/tau ratio improves the discrimination between sCJD and other dementias, especially AD, for which tau, p-tau and Aβ42 presented a partial overlap between groups. Second, the study population (n>2000 cases) provides statistical power and reliability to the findings. Third, tau and p-tau were measured using commercial immunoassays, which are standardized and worldwide used in clinical routine. Thus, the inclusion of the p-tau/tau ratio in diagnostic criteria of sCJD would be fairly affordable in terms of technical implementation.

In a more recent multinational study we determined CSF α-synuclein levels in a large cohort of CJD patients in comparison to a broad range of neurological and neurodegenerative conditions. Similar to p-tau/tau ratio, α-synuclein was exclusively altered in sCJD cases, allowing a valuable diagnostic discrimination from non- CJD cases with 94% sensitivity and 96% specificity [3]. These findings, obtained with an electrochemiluminiscent-based ELISA platform, were validated in two alternative cohorts of cases, highlighting the role of α-synuclein as a novel and reliable biomarker for sCJD. Yet, before this assay can be introduced into clinical practice, the robustness and precision of the test, along with an inter-laboratory standardization process should be performed according to standard operating procedures [4].

In addition to surrogate markers of the disease, in vitro assays mimicking the conversion of PrP, have been recently developed for the detection of PrPSc. One of the most common protein misfolding amplification systems is the real time quaking induced conversion (RT-QuIC), in which minuscule amounts of a PrPSc seed recruit molecules of recombinant PrP substrate inducing their conversion into an amyloid-like state that can be monitored in real time. Using this technique, we and others observed a sensitivity of 80-90% and a specificity of 99-100% in the identification of sCJD cases [5]. Studies on the stability of signal detection after define storage conditions as well as in ring trials, emphasize RT-QuIC as a very robust and reproducible test for sCJD diagnosis [6].

Besides their proved potential in the discrimination of sCJD cases from other dementias, an additional value of p-tau/tau ratio, α-synuclein and RT-QuIC is the discrimination of sCJD cases from other dementias displaying rapid progression course [2, 3, 5], underscoring the relevance of these biomarkers in the differentiation of neurodegenerative conditions with partial overlap on the clinical phenotype.

A paradox in the field is that, while CSF-based tests are gaining experimental momentum as their clinical accuracy allows almost full discrimination of sCJD cases, the current international diagnostic criteria only includes a positive 14-3-3 assay, along with a clinical duration leading to death in less than two years, in the recommended case definition for probable sCJD [7]. Therefore, in light of recent advances, we suggest that diagnostic criteria for sCJD should be revised in order to incorporate p-tau/tau ratio and RT-QuIC. In the same manner, quantitative detection of 14-3-3 protein [8] and α-synuclein [3] should be taken into account in the revision of the current diagnostic criteria. Altogether, these findings put CSF tests in the spotlight of sCJD clinical research becoming a crucial tool in the achievement of definite pre-mortem diagnosis, for which implementation of new diagnostic algorithms would be required.
